# Comparison of transesophageal echocardiography findings after different anticoagulation strategies in patients with atrial fibrillation: a systematic review and meta-analysis

**DOI:** 10.1186/s12872-019-1209-x

**Published:** 2019-11-26

**Authors:** Jian Yang, Xuan Zhang, Xi-ying Wang, Chi Zhang, Song-zan Chen, Shen-Jiang Hu

**Affiliations:** 1 Department of Cardiology, The First Affiliated Hospital, College of Medicine, Zhejiang University, Hangzhou, China; 2 College of Medicine, Zhejiang University, Hangzhou, China

**Keywords:** Transesophageal echocardiography, Left atrial thrombus, Anticoagulation, Atrial fibrillation

## Abstract

**Background:**

High risk of embolic events exists in both patients with chronic atrial fibrillation (AF) and patients in the perioperative period of ablation (effective treatment for AF). Therefore, anticoagulant therapy is important. Oral anticoagulants can be divided into two major categories: vitamin K antagonists (VKAs) and non-vitamin K antagonist oral anticoagulants (NOACs). VKAs, represented by warfarin, have been widely used as traditional anticoagulants, whereas NOACs have been used in clinical practice, but their anticoagulant effects and side effects are still the focus of research. We used a meta-analysis to compare the incidence of left atrial thrombi (LAT) between different anticoagulants.

**Methods:**

We searched PubMed, EMBASE, Web of Science, and the Cochrane Library databases for observational studies that compared the transesophageal echocardiography (TEE) findings for patients treated with NOACs and VKAs. The incidence of LAT and *dense spontaneous echocardiographic contrast (dense SEC)* were extracted as the basis of the meta-analysis.

**Results:**

Fifteen studies were included in the meta-analysis. We found that patients anticoagulated with NOACs and VKAs had similar incidence of LAT (OR = 0.74, 95%CI: 0.55–1.00). After excluding the heterogeneous article by sensitivity analysis, we found the incidence of LAT in patients anticoagulated with NOACs is lower than VKAs (OR = 0.59, 95%CI: 0.42–0.84). The results of subgroup analysis showed that the incidence of LAT among three types of NOACs have no significant difference (dabigatran vs. rivaroxaban, OR = 1.16 [0.75, 1.81]; rivaroxaban vs. apixaban, OR = 0.97 [0.54, 1.74]; dabigatran vs. apixaban, OR = 1.09 [0.55, 2.16]).

**Conclusion:**

Patients anticoagulated with NOACs may have lower incidence of LAT than VKAs. The incidence of LAT among different type of NOACs are similar.

## Background

Atrial fibrillation (AF) is the most commonly sustained cardiac arrhythmia, with a prevalence of 3% in adults [1]. The probability of stroke or heart failure is significantly increased in patients with AF [2]. Several studies have shown that 20–30% of ischemic stroke patients are simultaneously diagnosed with AF [3–5]. Research has previously shown that left atrial thrombi (LAT) are the main source of embolism and stroke [6]. Therefore, the formation of LAT must be prevented in patients with AF with either drug-induced anticoagulation or occlusion of the left atrial appendage [7]. Treatment of AF with ablation is the fundamental method of preventing LAT, but there is high risk of embolization during perioperative period. In general, drug anticoagulation is widely used as the first choice for thrombosis prevention. Currently, anticoagulant drugs can be divided into two categories: vitamin K antagonists (VKAs) and non-vitamin K antagonist oral anticoagulants (NOACs). VKAs, the anticoagulants first used in AF patients, can significantly reduce the risk of stroke and death [8]. However, the use of VKAs is limited by their shortcomings, such as the need to closely monitor the international normalized ratio (INR) and their susceptibility to genetic and environmental factors. In recent years, NOACs have been rapidly adopted as a substitute for VKAs in clinical practice [9]. The main advantage of NOACs is that the anticoagulant effect is predictable and the INR need not be monitored. However, whether NOACs are superior to VKAs has not been established. A meta-analysis showed that NOACs entailed a lower incidence of intracranial bleeding than VKAs, but a higher incidence of gastrointestinal bleeding [10]. Research on the anticoagulant effects and side effects of NOACs is still topical.

It has been reported that the annual risk of ischemic stroke in patients with AF alone is approximately 1.3% [11]. In a meta-analysis, the incidence of stroke was 0.35% in patients with AF undergoing anticoagulation drug therapy [12]. The guidelines suggest that oral anticoagulation should be started at least 3 weeks before cardioversion and continue for 4 weeks after it [13], and transesophageal echocardiography (TEE) can be used to rule out LAT before cardioversion [14]. In recent years, many studies have used TEE to compare the clinical results of different anticoagulants, including the incidence of LAT and the incidence of *dense spontaneous echocardiographic contrast (dense SEC)*. We conducted a meta-analysis to summarize these findings and to compare the incidence of LAT between patients anticoagulated with NOACs and VKAs.

## Methods

### Search strategy

In accordance with the Cochrane Handbook recommendations and the Preferred Reporting Items for Systematic Reviews and Meta-Analyses (PRISMA) guidelines [15, 16], we performed a systematic review of the literature and searched PubMed from the beginning of the database to March 2019. The Web of Science, EMBASE, and the Cochrane Library databases were also used for further searches. The search strings are reported in Additional file 1. The grey literature was not explored in this study.

Endnote X9 was used to eliminate duplicates. We browsed the titles and abstracts to identify observational studies that compared the incidence of LAT between different anticoagulants, and we read the full texts to eliminate studies that lacked relevant data.

### Document quality assessment

Two authors (XZ and JY) assessed the quality of the literature according to the Agency for Healthcare Research and Quality (AHRQ) scale independently. If there was disagreement, it was resolved by SJH.

### Data extraction

We extracted relevant data from these studies, including the number of included patients, the type of oral anticoagulants and the mode of administration, the INR for patients with VKAs, the ethnicity of patients, the incidence of LAT and dense SEC in TEE findings. The data for patients who took anticoagulants for < 3 weeks or who took them irregularly were excluded.

### Statistical analysis

We calculated the odds ratio (OR), with 95% confidence interval (CI), separately for each outcome and trial, and then pooled and compared the outcomes with a fixed-effects model. We assessed the heterogeneity of the data with the Cochran Q statistic and I^2^ test. Publication bias was tested with a funnel plot. A sensitivity analysis was used to further explore the heterogeneity of the included articles. We compared the pooled effect before and after deleting each document separately. If the effect changed, we considered that the article had a significant impact on the pooled effect. These documents were deleted and the others were analyzed further. All statistical analyses were performed with Review Manager (RevMan 5.3).

## Results

### Search results

**Fig. 1:**
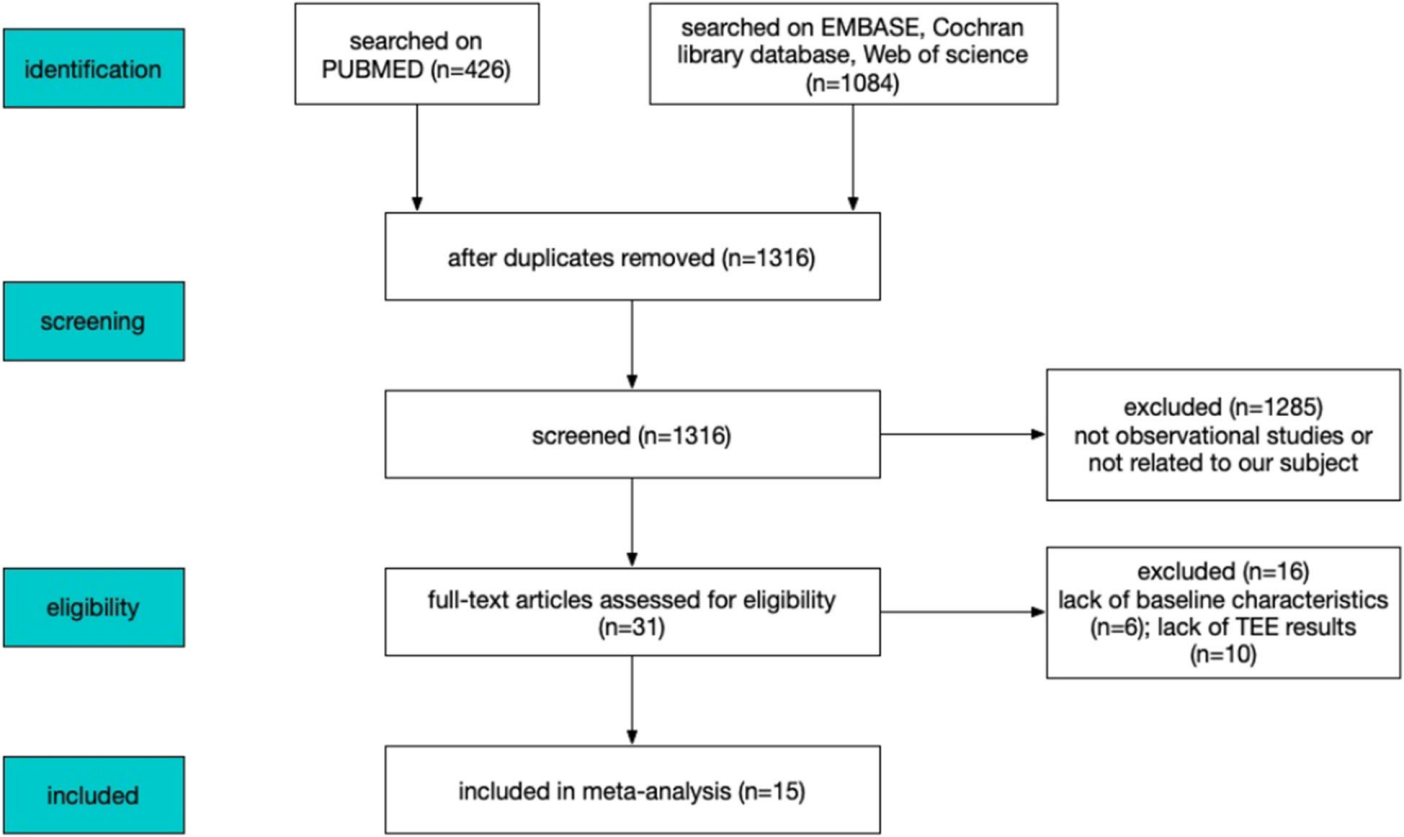
PRISMA flow diagram

We recovered 1510 articles with searches of the PubMed, Web of Science, EMBASE, and Cochrane Library databases. After we excluded duplicate articles with Endnote X9, 1316 articles remained. When we browsed the titles and abstracts, we excluded 1289 articles. The remaining 27 articles were observational studies comparing incidence of LAT between patients anticoagulated with VKAs and NOACs. After we read the full texts of these 27 articles, 12 were excluded because they lacked TEE data or patient information. The remaining 15 articles were included in this meta-analysis [17–31]. The PRISMA flow diagram is shown in Fig. 1. The AHRQ scale was used to assess the quality of the literature, as shown in Additional file 2. The INR of warfarin and dose profile were reported in Additional file 3.

### Meta-analysis

**Fig. 2:**
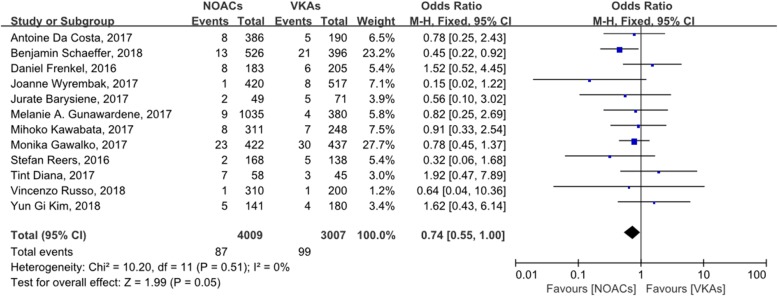
forest plot for LAT between NOACs and VKAs

**Fig. 3:**
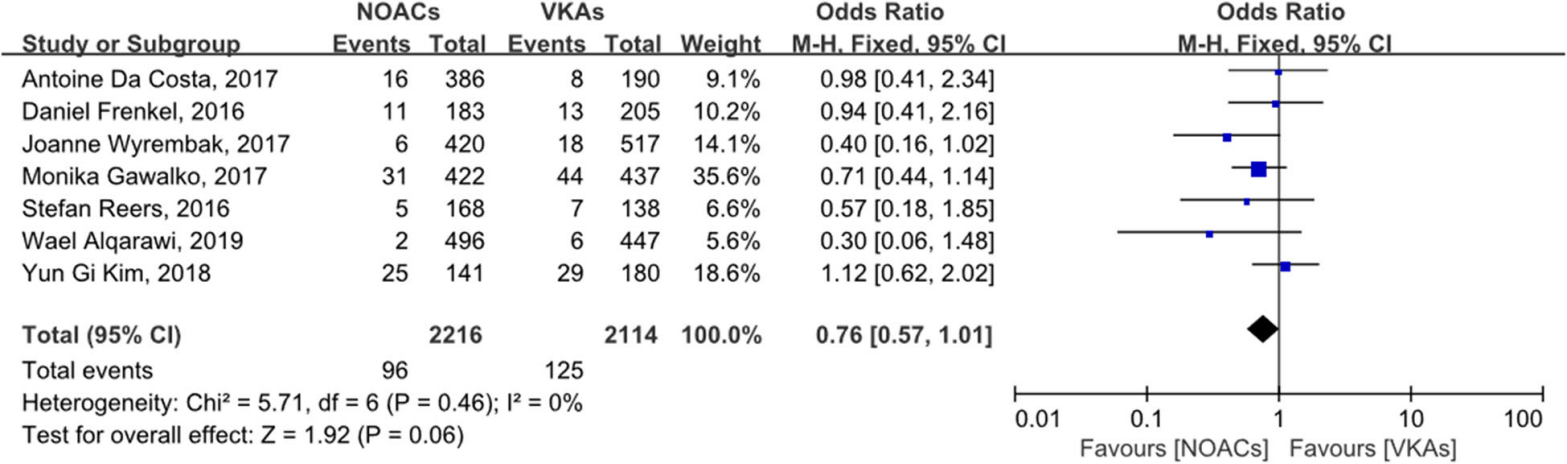
forest plot for LAT/dense SEC between NOACs and VKAs

Figure 2 shows the incidence of LAT in 7016 patients (4009 treated with NOACs; 3007 treated with VKAs) in 12 studies, with OR = 0.74 (95% CI: 0.55–1.00). Figure 3 shows the incidence of LAT and dense SEC in 4330 patients (2216 treated with NOACs; 2114 treated with VKAs) in seven studies, with OR = 0.76 (95% CI: 0.57–1.01). The incidence of LAT in patients treated with dabigatran, rivaroxaban, or apixaban is shown in Additional files 4, 5, or 6, respectively.

The Cochran Q statistic and I^2^ test suggested that there was no significant heterogeneity within the studies (*P* > 0.1, I^2^ = 0). A funnel plot (Additional file 7) did not suggest a high risk of publication bias. It is difficult to evaluate publication bias with a funnel plot in analyses that include < 10 studies.

### Sensitivity analysis

**Fig. 4:**
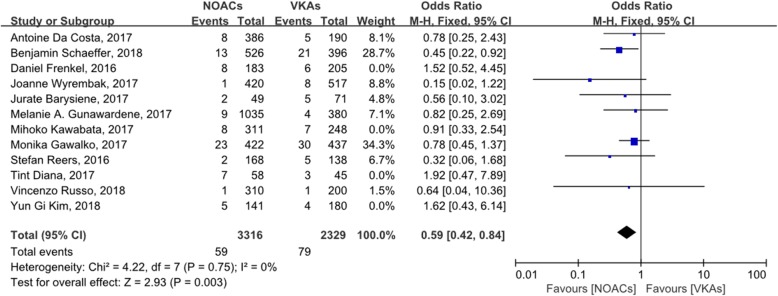
forest plot for LAT between NOACs and VKAs (after sensitivity analysis)

We analyzed the incidence of LAT in patients anticoagulated with NOACs or VKAs with a sensitivity analysis, which showed that four of the studies (Frenkel, 2016; Kawabata, 2017; Tint, 2017; and Kim, 2018) had a greater impact on the pooled effects than the other studies. When these four studies were deleted and the meta-analysis was performed again, the OR value was 0.59 (95% CI: 0.42–0.84) (Fig. 4). A further sensitivity analysis was performed on the remaining eight studies, and we found that the pooled effects did not change after the deletion of each individual study. So far the heterogeneity of the studies included in this group was considered low.

When we analyzed the incidence of LAT and dense SEC in the patients treated with NOACs or VKAs, a sensitivity analysis showed that two studies (Da Costa, 2017; Kim, 2018) had a greater impact on the pooled effects than the other studies. After we deleted these two studies, a sensitivity analysis of the remaining studies was performed, and another study (Wyrembak, 2017) was found to have a greater impact on the pooled effects than the other studies. Therefore, we believe that the heterogeneity among these studies was very large and the reliability of this result was considered low.

A sensitivity analysis of the articles that examined the three individual NOACs showed that none influenced the pooled effects significantly, and the articles included in the three analyses were considered to be less heterogeneous than those used in the overall analysis. Therefore, the results were reliable.

### Subgroup analysis

**Fig. 5:**
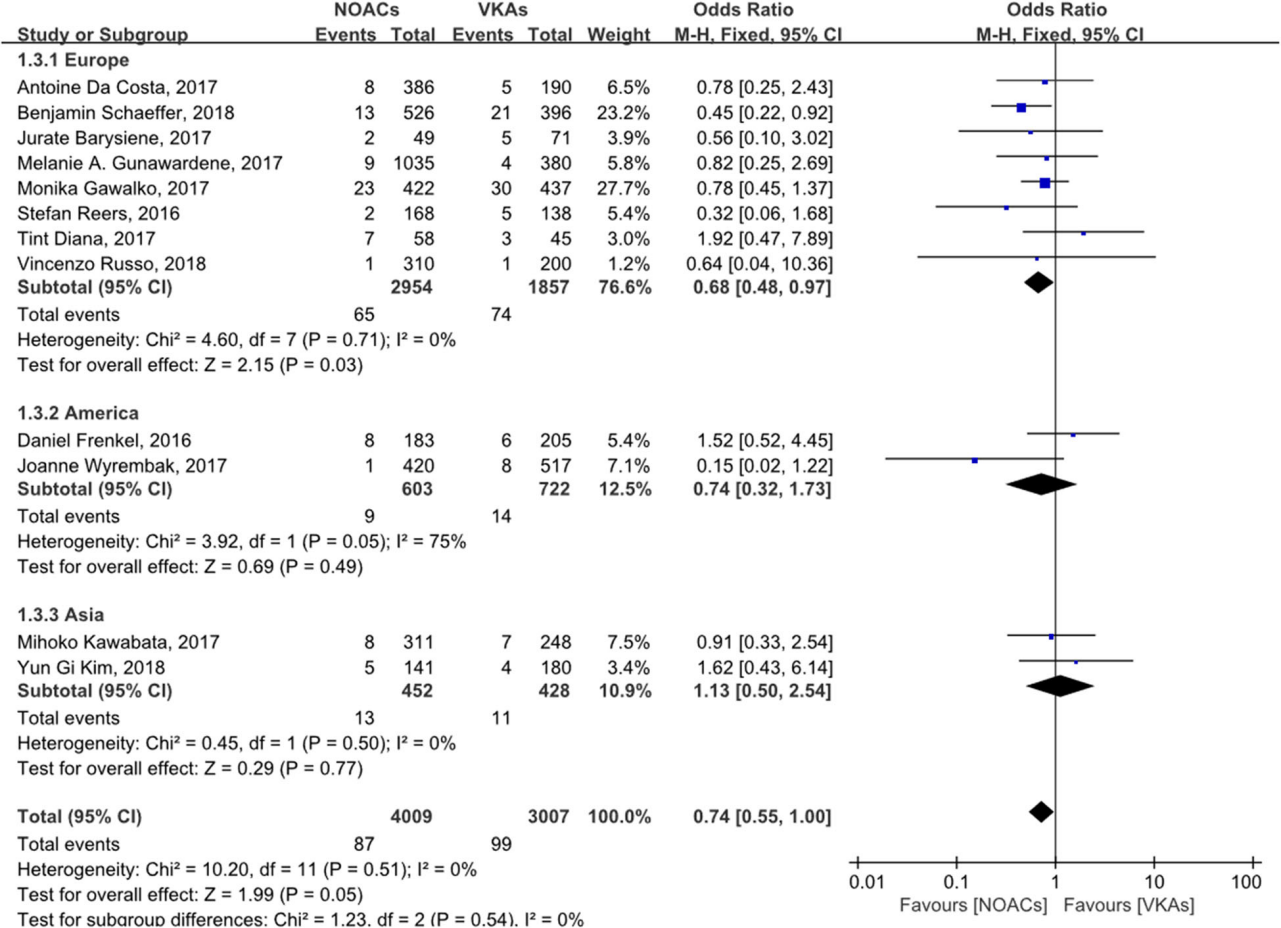
subgroup analysis of races

A subgroup analysis of races showed that for Europeans, the incidence of LAT in patients with NOACs is lower than VKAs (OR: 0.68, 95%CI: 0.48–0.97), while there was no such significant difference among Asians or Americans (Fig. 5).

**Fig. 6:**
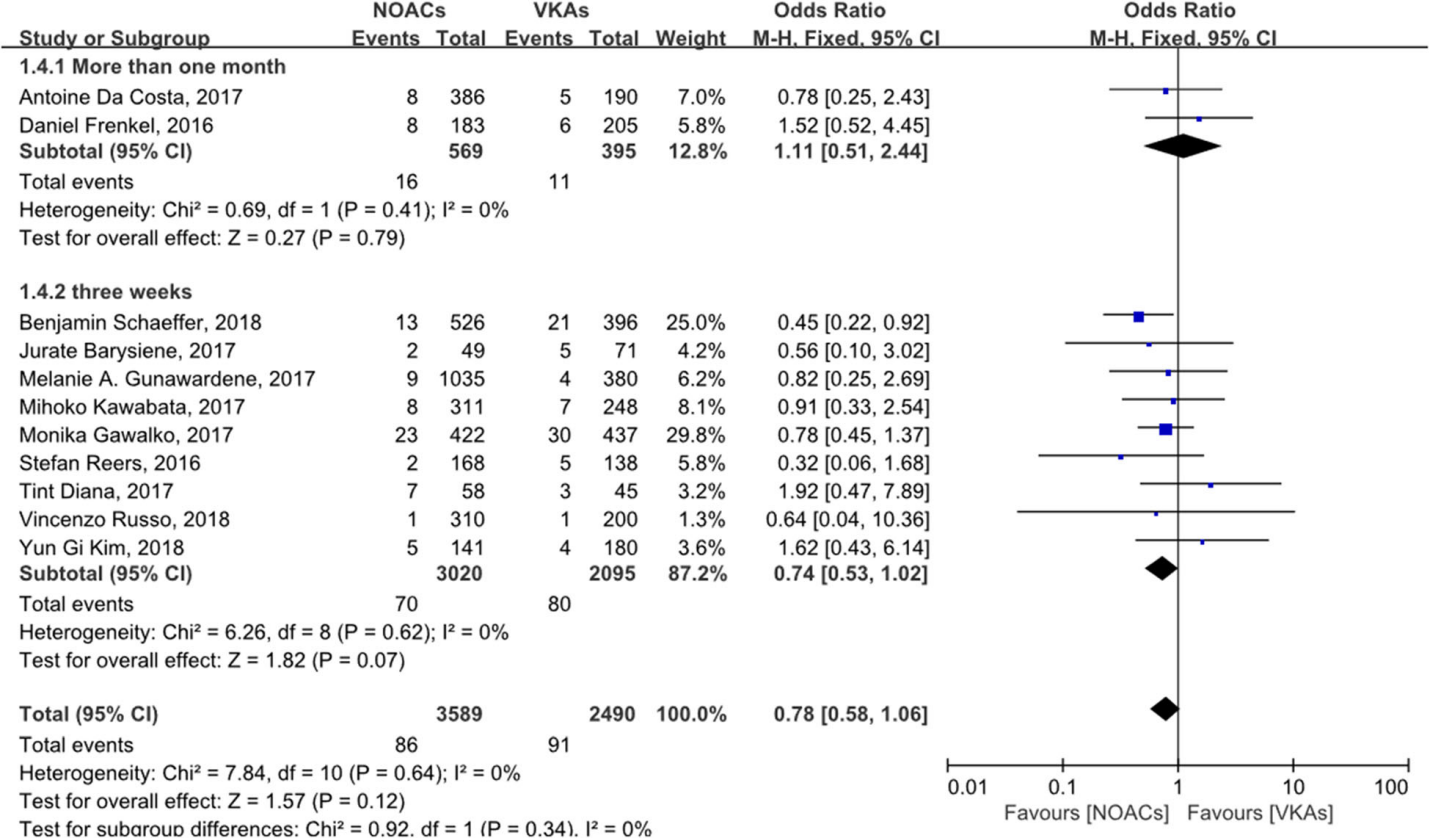
subgroup analysis of anticoagulation time

A subgroup analysis of the anticoagulation time showed that the incidence of LAT in patients with NOACs and VKAs were similar during anticoagulation for 3 weeks, which is according to the guideline, or during anticoagulation for more than 1 month (Fig. 6).

## Discussion

In this study, we compared the incidence of LAT in patients with AF anticoagulated with NOACs or VKAs, and we detected no significant difference between NOACs and VKAs (OR: 0.74, 95% CI: 0.55–1.00). Both the Q statistic and I^2^ test suggested that there was no significant heterogeneity in the included studies (*P* = 0.51, I^2^ = 0), and a funnel plot did not suggest significant publication bias. To further analyze the heterogeneity between the studies included, we performed a sensitivity analysis and found that four studies significantly influenced the pooled effects, indicating that they differed significantly from the other studies. When we omitted these studies from the meta-analysis, the incidence of LAT in patients anticoagulated with NOACs is lower than VKAs (OR: 0.59, 95% CI: 0.42–0.84). The sensitivity analysis was repeated on the remaining studies, and no significant heterogeneity was detected. Therefore, we believe that the results of this analysis were reliable relatively.

Dense SEC predicts thrombosis, and the presence of LAT and/or dense SEC can be used as an indicator of left atrial abnormalities. Therefore, we also analyzed the incidence of LAT or dense SEC between patients with NOACs and VKAs, but detected no significant difference between them (OR: 0.76, 95% CI: 0.57–1.01). Both the Q statistic and I^2^ test indicated that there was no significant heterogeneity in the studies included (*P* = 0.46, I^2^ = 0). Because fewer than 10 studies were included in this meta-analysis, a funnel plot could not be used to evaluate the publication bias. In the sensitivity analysis, two studies had a significant impact on the pooled effects. After these two were omitted, the remaining five studies were analyzed for sensitivity, and another study was found to significantly affect the pooled effects. Therefore, we believe that the studies included in this analysis were highly heterogeneous and that the results obtained were unreliable.

Dabigatran, rivaroxaban, and apixaban are commonly used NOACs, so we analyzed the effects of the three drugs. There was no significant difference among the three medications in the incidence of LAT. However, because of the small scale of the data, we did not perform a LAT/dense SEC analysis. Considering the inevitable differences in research methods used in the studies, we conducted a subgroup analysis for further discussion. A subgroup analysis of races suggested that NOACs are more effective than VKAs in Europeans (OR: 0.68, 95%CI: 0.48–0.97), whereas NOACs and VKAs are similarly effective among Asians. However, studies of American population were highly heterogeneous (*P* = 0.05, I2 = 75%) and there are differences in the characteristics of patients using VKAs and NOACs due to the circumstances of each country, so we could draw no convincing conclusion. In fact, the number of studies and their included patients for Asian or American are far less than European. Multicenter randomized controlled trials with large sample size and standardized protocol may be required to analyze the difference among races.

In all included studies, some followed the guideline to start anticoagulation therapy 3 weeks before screening LAT, whereas others administered anticoagulation for more than 1 month. A subgroup analysis of this factor detected no statistically significant difference in the incidence of LAT between patients anticoagulated with NOACs and VKAs over time. Despite this, the trend of a forest plot seemed to suggest that NOACs are more effective for short-term anticoagulation.

### Limitation

The results of this study suggest that the incidence of LAT in patients anticoagulated with NOACs may be lower than VKAs. Because only observational studies were included in this analysis, there was inevitably large heterogeneity, which challenges the credibility of our conclusions. To minimize the effects of heterogeneity, we used sensitivity analyses and subgroup analyses, which reduced the heterogeneity to some extent. However, the heterogeneity caused by the doctors’ subjective choice of medication, the patients’ subjective intentions, the researchers’ preferences, and the differences in the patients’ baseline data between the two groups are important limitations of this analysis. The INR of patients with VKAs and the dosage of patients anticoagulated with NOACs was also different among included studies, which may influence the effect of anticoagulation to a certain extent. Therefore, the conclusions drawn in this analysis should be confirmed with a randomized controlled trial to ensure greater reliability. For patients with severe renal dysfunction, NOACs are not recommended. Therefore, for retrospective studies, renal function affects medication choices and furtherly affects research results. Unfortunately, the included studies did not give the data about patients’ renal function, which is a limitation in this meta-analysis. Last but not least, AF caused by valvular heart disease should be considered when discussing anticoagulation even though all included studies analyzed non-valvular AF only. The guideline [13] suggests that VKAs remains the mainstay of anticoagulation for AF patients with valvular heart disease, while the safety and efficacy of NOACs has not been evaluated and should be studied.

## Conclusion

This meta-analysis assessed the incidence of LAT in patients with AF treated with different anticoagulation strategies. The incidence of LAT was lower in patients anticoagulated with NOACs than in those anticoagulated with VKAs (OR: 0.59, 95%CI: 0.42–0.84). There was no significant difference in incidence of LAT when anticoagulated with dabigatran, rivaroxaban, or apixaban.

## Supplementary information

**Additional file 1:** Search string.

**Additional file 2:** AHRQ scale.

**Additional file 3:** Dose profile of anticoagulants.

**Additional file 4:** Forest plot for LAT/dense SEC between dabigatran and rivaroxaban.

**Additional file 5:** Forest plot for LAT/dense SEC between rivaroxaban and apixaban.

**Additional file 6:** Forest plot for LAT/dense SEC between dabigatran and apixaban.

**Additional file 7:** Funnel plot for LAT between NOACs and VKAs.

## Data Availability

The datasets generated and analyzed during the current study are available from the corresponding author on reasonable request.

## Abbreviations

AF: Atrial fibrillation
AHRQ: Agency for healthcare research and quality
CI: Confidence interval
dense SEC: Dense spontaneous echocardiographic contrast
INR: International normalized ratio
LAT: Left atrial thrombi
NOACs: Non-vitamin K antagonist oral anticoagulants
OR: Odds ratio
PRISMA: Preferred reporting items for systematic reviews and meta-analyses
TEE: Transesophageal echocardiography
VKAs: Vitamin K antagonists

## References

[1] Bjorck S, Palaszewki B, Friberg L (2013). Atrial fibrillation, stroke risk, and warfarin in therapy revisited: a population-based study. Stroke.

[2] Stewart S, Hart CL, Hole DJ (2002). A population-based study of the long-term risks associated with atrial fibrillation: 20-year follow-up of the Renfrew/Paisley study. Am J Med.

[3] Kishore A, Vail A, Majid A (2014). Detection of atrial fibrillation after ischemic stroke or transient ischemic attack: a systematic review and meta-analysis. Stroke.

[4] Henriksson KM, Farahmand B, Asberg S (2012). Comparison of cardiovascular risk factors and survival in patients with ischemic or hemorrhagic stroke. Int J Stroke.

[5] Grond M, Jauss M, Hamann G (2013). Improved detection of silent atrial fibrillation using 72-hour Holter ECG in patients with ischemic stroke: a prospective multicenter cohort study. Stroke.

[6] Zotova IV, Zateishchikov DA, Sidorenko BA (2013). Mechanisms of development of thromboembolic complications in patients with atrial fibrillation. Kardiologiia.

[7] Holmes DR, Doshi SK, Kar S (2015). Left atrial appendage closure as an alternative to warfarin for stroke prevention in atrial fibrillation: a patient-level meta-analysis. J Am Coll Cardiol.

[8] Hart RG, Pearce LA, Aguilar MI (2007). Meta-analysis: antithrombotic therapy to prevent stroke in patients who have nonvalvular atrial fibrillation. Ann Intern Med.

[9] Olesen JB, Sorensen R, Hansen ML (2015). Non-vitamin K antagonist oral anticoagulation agents in anticoagulant naïve atrial fibrillation patients: Danish nationwide descriptive data 2011-2013. Europace.

[10] Ruff CT, Giugliano RP, Braunwald E (2014). Comparison of the efficacy and safety of new oral anticoagulants with warfarin in patients with atrial fibrillation: a meta-analysis of randomized trials. Lancet.

[11] Ali S, Hong M, Antezano ES (2006). Evaluation and management of atrial fibrillation. Cardiovasc Hematol Disord Drug Targets.

[12] Telles Garcia N, Dahal K, Kocherla C (2018). Non-vitamin K antagonists oral anticoagulants are as safe and effective as warfarin for cardioversion of atrial fibrillation: a systematic review and meta-analysis. Int J Cardiol.

[13] Kirchhof P, Benussi S, Kotecha D (2016). 2016 ESC guidelines for the management of atrial fibrillation developed in collaboration with EACTS. Europace.

[14] Blackshear JL, Odell JA (1996). Appendage obliteration to reduce stroke in cardiac surgical patients with atrial fibrillation. Ann Tohrac Surg.

[15] Higgins JPT, Green S (2011). Cochrane handbook for systematic reviews of interventions.

[16] Moher D, Altman DG, Liberati A, Tetzlaff J (2011). PRISMA statement. Epidemiology.

[17] Alqarawi W, Birnie DH, Spence S (2019). Prevalence of left atrial appendage thrombus detected by transoesophageal echocardiography before catheter ablation of atrial fibrillation in patients anticoagulated with non-vitamin K antagonist oral anticoagulants. Europace.

[18] Schaeffer B, Ruden L, Salzbrunn T (2018). Incidence of intracardiac thrombus formation prior to electrical cardioversion in respect to the mode of oral anticoagulation. J Cardiovasc Electrophysiol.

[19] Kim YG, Chi JI, Kim MN (2018). Non-vitamin K antagonist oral anticoagulants versus warfarin for the prevention of spontaneous echo-contrast and thrombus in patients with atrial fibrillation or flutter undergoing cardioversion: a trans-esophageal echocardiography study. PLoS One.

[20] Russo V, Rago A, Papa AA (2018). Efficacy and safety of dabigatran in patients with atrial fibrillation scheduled for transoesophageal echocardiogram-guided direct electrical current cardioversion: a prospective propensity score-matched cohort study. J Thromb Thrombolysis.

[21] Da Costa A, Delolme C, Guichard JB (2017). Comparison of prevalence and management of left atrial appendage thrombi under old and new anticoagulants prior to left atrial catheter ablation. Am Heart J.

[22] Gawalko M, Kaplon-Cieslicka A, Budnik M (2017). Comparison of different oral anticoagulant regimens in patients with atrial fibrillation undergoing ablation or cardioversion. Pol Arch Intern Med.

[23] Tint D, Petris AO, Pop I (2017). Vitamin K antagonists versus novel oral anticoagulants for elective electrical cardioversion of atrial fibrillation. Am J Ther.

[24] Gunawardene MA, Dickow J, Schaeffer BN (2017). Risk stratification of patients with left atrial appendage thrombus prior to catheter ablation of atrial fibrillation: an approach towards an individualized use of transesophageal echocardiography. J Cardiovasc Electrophysiol.

[25] Barysiene J, Zebrauskaite A, Petrikonyte D (2017). Findings of transoesophageal echocardiogram in appropriately anticoagulated patients with persistent atrial fibrillation prior to planned cardioversion. BMC Cardiovasc Disord.

[26] Kawabata M, Goya M, Sasaki T (2017). Left atrial appendage thrombi formation in Japanese non-valvular atrial fibrillation patients during anticoagulation therapy - warfarin vs. direct oral anticoagulants. Circ J.

[27] Wyrembak J, Campbell KB, Steinberg BA (2017). Incidence and predictors of left atrial appendage thrombus in patients treated with nonvitamin K oral anticoagulants versus warfarin before catheter ablation for atrial fibrillation. Am J Cardiol.

[28] Reers S, Agdirlioglu T, Kellner M (2016). Incidence of left atrial abnormalities under treatment with dabigatran, rivaroxaban, and vitamin K antagonists. Eur J Med Res.

[29] Frenkel D, D’Amato SA, AI-Kazaz M (2016). Prevalence of left atrial thrombus detection by transesophageal echocardiography: a comparison of continuous non-vitamin K antagonist oral anticoagulant versus warfarin therapy in patients undergoing catheter ablation for atrial fibrillation. JACC Clin Electrophysiol.

[30] Wu M, Gabriels J, Khan M (2018). Left atrial thrombus and dense spontaneous echocardiographic contrast in patients on continuous direct oral anticoagulant therapy undergoing catheter ablation of atrial fibrillation: comparison of dabigatran, rivaroxaban, and apixaban. Heart Rhythm.

[31] Bertaglia E, Anselmino M, Zorzi A (2017). NOACs and atrial fibrillation: incidence and predictors of left atrial thrombus in the real world. Int J Cardiol.

