# Supplementary material for: Comparison of transesophageal echocardiography findings after different anticoagulation strategies in patients with atrial fibrillation: a systematic review and meta-analysis
Source: BMC Cardiovasc Disord. 2019 Nov 26;19:261. doi: 10.1186/s12872-019-1209-x (PMC6878716; doi:10.1186/s12872-019-1209-x)
Supplement: Supplementary file 1 — Additional file 1: Search string. [file 12872_2019_1209_MOESM1_ESM.docx]

Additional file 1. Search string

The search strings in different databases are showed as follows.

Pubmed: (NOAC [all fields] OR NOACs [all fields] OR anticoagulant [all fields] OR anticoagulants [all fields]) AND (fibrillation [all fields] OR fibrillations [all fields]) AND (trans-esophageal echocardiography [all fields] OR transesophageal echocardiography [all fields]) AND (thrombosis [all fields] OR thrombi [all fields] OR thrombus [all fields]).

Web of science: TS=(NOAC*) AND TS=(fibrillation*) AND TS=(transesophageal* echocardiography*) AND TS=(thrombosis*).

EMBASE: NOAC* AND fibrillation* AND transesophageal echocardiography AND thrombosis.

Cochrane Library database: NOACs (all text) AND fibrillation (all text) AND transesophageal echocardiography (all text) AND thrombosis (all text).
