# Supplementary material for: Comparison of transesophageal echocardiography findings after different anticoagulation strategies in patients with atrial fibrillation: a systematic review and meta-analysis
Source: BMC Cardiovasc Disord. 2019 Nov 26;19:261. doi: 10.1186/s12872-019-1209-x (PMC6878716; doi:10.1186/s12872-019-1209-x)
Supplement: Supplementary file 2 — Additional file 2: AHRQ scale. [file 12872_2019_1209_MOESM2_ESM.docx]

Additional file 2. AHRQ scale

|  | 1) Define the source of information (survey, record review) | 2) List inclusion and exclusion criteria for exposed and unexposed subjects (cases and controls) or refer to previous publications | 3) Indicate time period used for identifying patients | 4) Indicate whether or not subjects were consecutive if not population-based | 5) Indicate if evaluators of subjective components of study were masked to other aspects of the status of the participants | 6) Describe any assessments undertaken for quality assurance purposes (e.g., test/retest of primary outcome measurements) | 7) Explain any patient exclusions from analysis | 8) Describe how confounding was assessed and/or controlled. | 9) If applicable, explain how missing data were handled in the analysis | 10) Summarize patient response rates and completeness of data collection | 11) Clarify what follow-up, if any, was expected and the percentage of patients for which incomplete data or follow-up was obtained |
| --- | --- | --- | --- | --- | --- | --- | --- | --- | --- | --- | --- |
| Wael Alqarawi, 2019 | + | + | + | + | + | + | + |  | + | + | + |
| Benjamin Schaeffer, 2018 | + | + | + | + | + | + | + | - | + | + | + |
| Yun Gi Kim, 2018 | + | + | + | + | + | + | + | + | - | + | + |
| Vincenzo Russo, 2018 | + | + | + | + | + | - | + | - | + | + | + |
| Antoine Da Costa, 2017 | + | + | + | + | + | + | + |  | + | + | + |
| Monika Gawalko, 2017 | + | + | + | + | - | + | + | + | + | + | + |
| Tint Diana, 2017 | + | + | + | + | + | - | + | + | + | + | + |
| Melanie A. Gunawardene, 2017 | + | + | + | + | + | + | + |  | + | + | + |
| Jurate Barysiene, 2017 | + | + | + | + | + | + | + |  | + | + | + |
| Mihoko Kawabata, 2017 | + | + | + | + | + | + | + |  | + | + | + |
| Joanne Wyrembak, 2017 | + | + | + | + | + | + | + | - | + | + | + |
| Stefan Reers, 2016 | + | + | + | + | + | + | + | + | + | + | + |
| Daniel Frenkel, 2016 | + | + | + | + | + | + | + |  | + | + | + |
| Michael Wu, 2018 | + | + | + | + | + | + | + | - | + | + | + |
| Emanuele Bertaglia, 2017 | + | + | + | + | + | + | + | - | + | + | + |

Risk of bias summary: review authors' judgements about each risk of bias item for each included study. For interpretation: + indicates low risk of bias, - indicates high risk of bias, and no specification indicates unclear or unknown risk of bias.
