# Supplementary material for: Comparison of transesophageal echocardiography findings after different anticoagulation strategies in patients with atrial fibrillation: a systematic review and meta-analysis
Source: BMC Cardiovasc Disord. 2019 Nov 26;19:261. doi: 10.1186/s12872-019-1209-x (PMC6878716; doi:10.1186/s12872-019-1209-x)
Supplement: Supplementary file 3 — Additional file 3: Dose profile of anticoagulants. [file 12872_2019_1209_MOESM3_ESM.docx]

Additional file 3. dose profile of anticoagulants

|  | Warfarin (INR) | Rivaroxaban (dose) | Dabigatran (dose) | Apixaban (dose) |
| --- | --- | --- | --- | --- |
| Wael Alqarawi, 2019 | At least 2 | Not reported | Not reported | Not reported |
| Benjamin Schaeffer, 2018 | 2.41±0.6 | 3.6% reduced | 2.8% reduced | 5% reduced |
| Yun Gi Kim, 2018 | 2.2±0.8 | 15mg or 10mg qd | 110mg bid | 2,5mg bid |
| Vincenzo Russo, 2018 | 2.1 | / | 150mg or 110mg bid | / |
| Antoine Da Costa, 2017 | 3~4 | 15-20mg qd | 110mg or 150 mg bid | 2.5mg or 5mg bid |
| Monika Gawalko, 2017 | Not reported | 20mg or 15mg qd | 150mg or 110mg bid | / |
| Tint Diana, 2017 | 2.5±0.6 | standard | standard | standard |
| Melanie A. Gunawardene, 2017 | 2.0~3.0 | Not reported | Not reported | Not reported |
| Jurate Barysiene, 2017 | 2.0~3.0 | standard | standard | standard |
| Mihoko Kawabata, 2017 | 1.6~2.6 (59.5% patients achieved) | 15mg or 10mg qd | 150mg or 110mg bid | 5mg or 2.5mg bid |
| Joanne Wyrembak, 2017 | 2.3±0.6 | 20mg or 10mg qd | 150mg bid | 5mg or 2.5mg bid |
| Stefan Reers, 2016 | 2.0~3.0 (67% patients achieved) | 15mg qd | 110mg bid | / |
| Daniel Frenkel, 2016 | Not reported | Not reported | Not reported | Not reported |
| Michael Wu, 2018 | / | standard | standard | standard |
| Emanuele Bertaglia, 2017 | 2.5~3.5 | standard | standard | standard |

Management of anticoagulants in included studies.

INR: international normalized ratio; Not reported: not mentioned in the article; “/”: the study did not set this group; standard/reduced: the article states that the standard/reduced dose is used but the specific value is not stated.
