# Supplementary material for: Comparison of transesophageal echocardiography findings after different anticoagulation strategies in patients with atrial fibrillation: a systematic review and meta-analysis
Source: BMC Cardiovasc Disord. 2019 Nov 26;19:261. doi: 10.1186/s12872-019-1209-x (PMC6878716; doi:10.1186/s12872-019-1209-x)
Supplement: Supplementary file 4 — Additional file 4: Forest plot for LAT/dense SEC between dabigatran and rivaroxaban. [file 12872_2019_1209_MOESM4_ESM.docx]

Additional file 4. forest plot for LAT/dense SEC between dabigatran and rivaroxaban


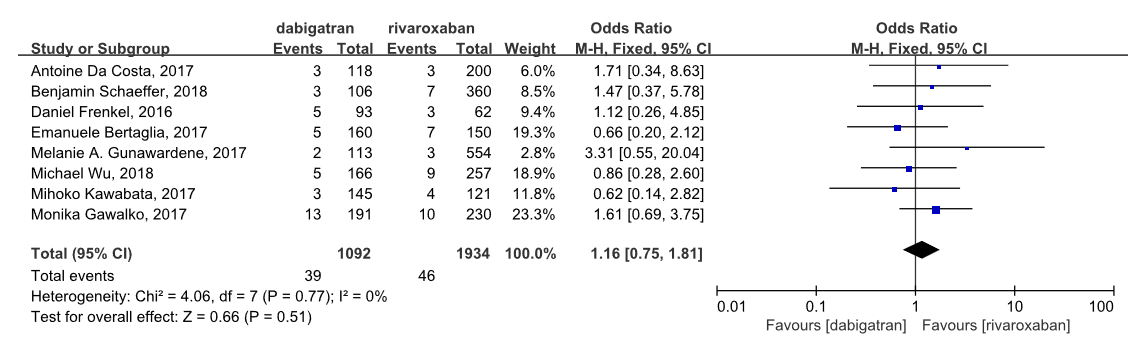


Comparison between dabigatran and rivaroxaban on LAT/dense SEC. No statistical difference was showed (OR: 1.16, 95% CI: 0.75–1.81).
