# Supplementary material for: Comparison of transesophageal echocardiography findings after different anticoagulation strategies in patients with atrial fibrillation: a systematic review and meta-analysis
Source: BMC Cardiovasc Disord. 2019 Nov 26;19:261. doi: 10.1186/s12872-019-1209-x (PMC6878716; doi:10.1186/s12872-019-1209-x)
Supplement: Supplementary file 6 — Additional file 6: Forest plot for LAT/dense SEC between dabigatran and apixaban. [file 12872_2019_1209_MOESM6_ESM.docx]

Additional file 6. forest plot for LAT/dense SEC between dabigatran and apixaban


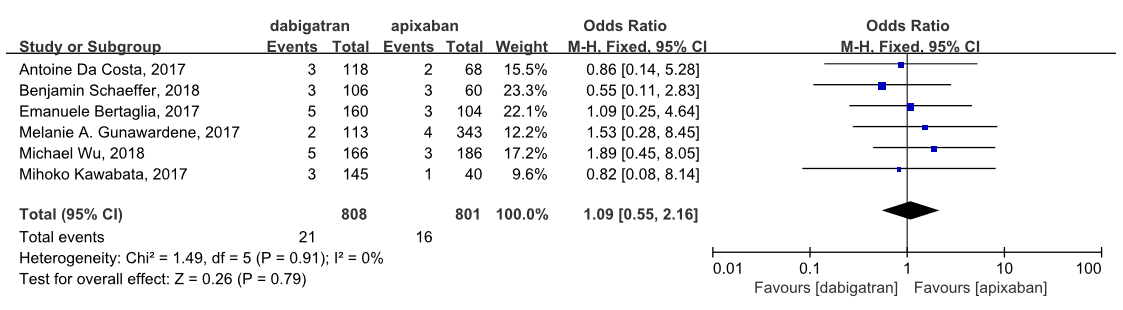


Comparison between dabigatran and apixaban on LAT/dense SEC. No statistical difference was showed (OR: 1.09, 95% CI: 0.55–2.16).
