# Supplementary material for: Comparison of transesophageal echocardiography findings after different anticoagulation strategies in patients with atrial fibrillation: a systematic review and meta-analysis
Source: BMC Cardiovasc Disord. 2019 Nov 26;19:261. doi: 10.1186/s12872-019-1209-x (PMC6878716; doi:10.1186/s12872-019-1209-x)
Supplement: Supplementary file 7 — Additional file 7: Funnel plot for LAT between NOACs and VKAs. [file 12872_2019_1209_MOESM7_ESM.docx]

Additional file 7. Funnel plot for LAT between NOACs and VKAs


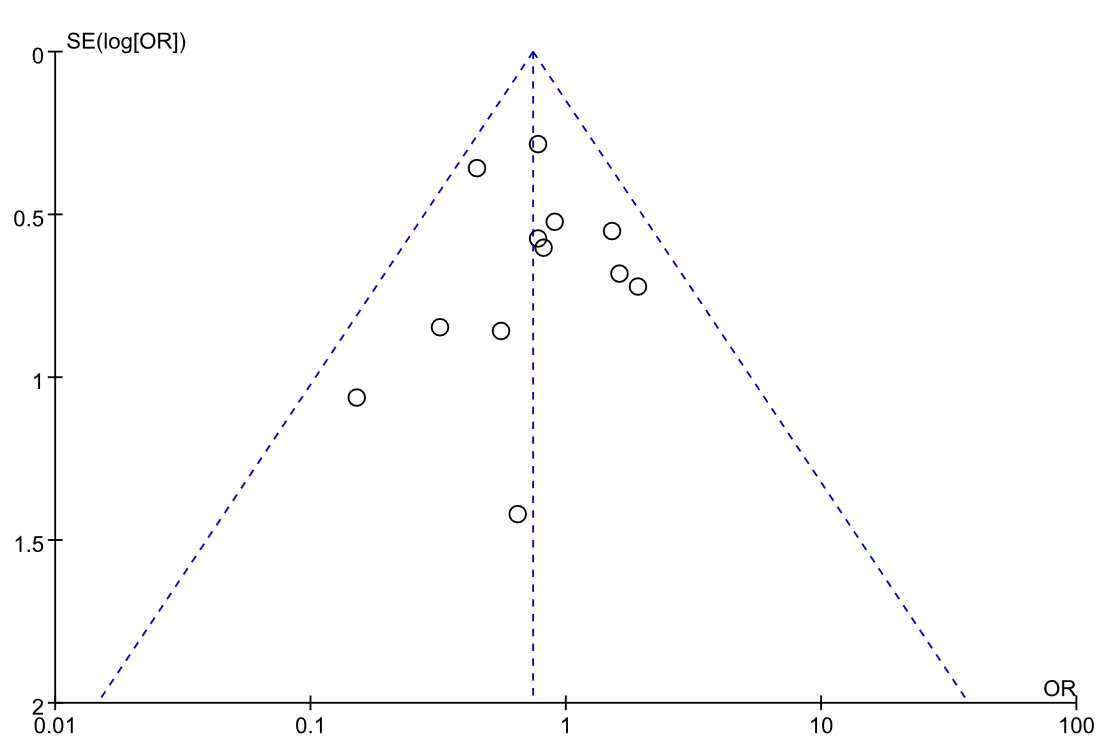


No significant publication bias was observed.
